# The novel protective role of P27 in MLN4924-treated gastric cancer cells

**DOI:** 10.1038/cddis.2015.215

**Published:** 2015-08-27

**Authors:** Q Zhang, D Hou, Z Luo, P Chen, B Lv, L Wu, Y Ma, Y Chu, H Liu, F Liu, S Yu, J Zhang, D Yang, J Liu

**Affiliations:** 1 Department of Digestive Diseases, Huashan Hospital, Fudan University, Shanghai, China; 2 Institutes of Biomedical Sciences of Shanghai Medical School, Fudan University, Shanghai, China; 3 Department of Immunology, Shanghai Medical College, Fudan University, Shanghai, China; 4 The Third Department of Hepatic Surgery, Eastern Hepatobiliary Surgery Hospital, Second Military Medical University, Shanghai, China

## Abstract

The tumor-suppressor gene cyclin-dependent kinase inhibitor 1B (P27) is downregulated in gastric cancer cells mainly through proteolytic degradation mediated by the SKP-Cullin1-F-Box (SCF) complex. But the correlation between its downregulation and gastric cancer prognosis still remains indefinite. MLN4924, an anti-tumor agent, which suppresses the SCF complex by inhibiting Cullin1 neddylation, emerges as a promising tool to elucidate its functions in gastric cancer cells. In this study, MLN4924 induced significant growth inhibition of gastric cancer cells in a dose-dependent manner, along with the simultaneous accumulation of P27 and cell cycle abnormalities such as G2/M arrest. Importantly, we found that P27 silencing in MLN4924-treated cells resulted in an enhancement of growth inhibition both *in vitro* and *in vivo*. Mechanism analysis revealed the antagonism effects of antioxidants to this excess apoptosis, suggesting reactive oxygen species (ROS) overproduction especially in the mitochondria was the principal cause of the augmentation. Moreover, the robust ROS attacked the mitochondria to initiate collapse of the mitochondrial membrane permeability and the exportation of apoptosis-inducing factor (AIF), IAP-binding mitochondrial protein (SMAC/DIABLO) and cytochrome c. Finally, we also found that P27 knockdown affected the expression profile of several critical BH3 family members to amplify the mitochondrial dysfunction and apoptosis. In summary, our findings unveiled a protective role of P27 by maintaining mitochondrial membrane permeability in MLN4924-treated gastric cancer cells, and therefore highlighted the potential combination of MLN4924 with P27 inhibition to improve its therapeutic efficacy.

Gastric cancer is a heavy burden to public health as its overall mortality ranked third in cancer-related deaths worldwide in 2012.^1^ Its asymptomatic progression in early stages often leads to delayed diagnosis and hence the poor prognosis of patients. Given the previous studies that demonstrated that S-phase kinase-associated protein 2 (SKP2) and Cullin1 (CUL1) were tightly connected with the progression of gastric cancer,^2, 3, 4^ the neddylation inhibitor MLN4924, specifically suppressing the functions of the SKP2-CUL1-F-box (SCF) complexes by blocking the CUL1 neddylation, is therefore a promising drug for the chemotherapy of gastric cancer.^5^

Cyclin-dependent kinase inhibitor 1B (P27) from the Cip/Kip family is a well-defined substrate for the ubiquitin ligase activity of SCF complex,^6, 7, 8^ and has a central role in cell cycle regulation.^9^ By getting incorporated into the CDK4/6-Cyclin D complex or the CDK2-Cyclin E/A complex, P27 restrains the G1 phase initiation and G1/S transition.^10, 11, 12, 13^ *In vivo* studies which reported organ hyperplasia in P27−/− mice confirmed the function of P27 as a tumor-suppressor gene with haplo-insufficient effects.^14, 15, 16, 17^ Numerous pathological studies have verified the P27 downregulation in various tumors, including breast,^18^ colon,^19^ lung,^20^ liver^21^ and stomach.^22^ In contrast to the positive correlations between P27 decline and tumor progression in other organs, the relationship of P27 expression and the prognosis of gastric cancer still remains controversial.^23, 24, 25^

Different from other tumor-suppressor genes, the decreased protein level of P27 in tumors was mainly due to the degradation mediated by the over-activated SCF complexes rather than DNA mutations.^16, 26^ In this study, we applied MLN4924 to investigate the functions of P27 as an important substrate of SCF complex in gastric cancer cells. Upon the cytotoxicity of MLN4924, P27 silencing in treated cells increased the mitochondrial reactive oxygen species (ROS) overproduction which initiated the synergic responses of BH3 family members and the release of mitochondrial apoptotic substances. These effects stimulated the mitochondrial membrane permeability collapse and activated intrinsic apoptotic signals to enhance the MLN4924-induced apoptosis. Our findings for the first time revealed the protective role of P27 in MLN4924-treated gastric cancer cells. Moreover, it emphasized the involvement of P27 in maintaining mitochondrial membrane permeability and the potential combination of MLN4924 with P27 inhibition to improve its therapeutic efficacy.

## Results

### The growth of gastric cancer cells was suppressed by the neddylation inhibitor MLN4924

**Figure 1:**
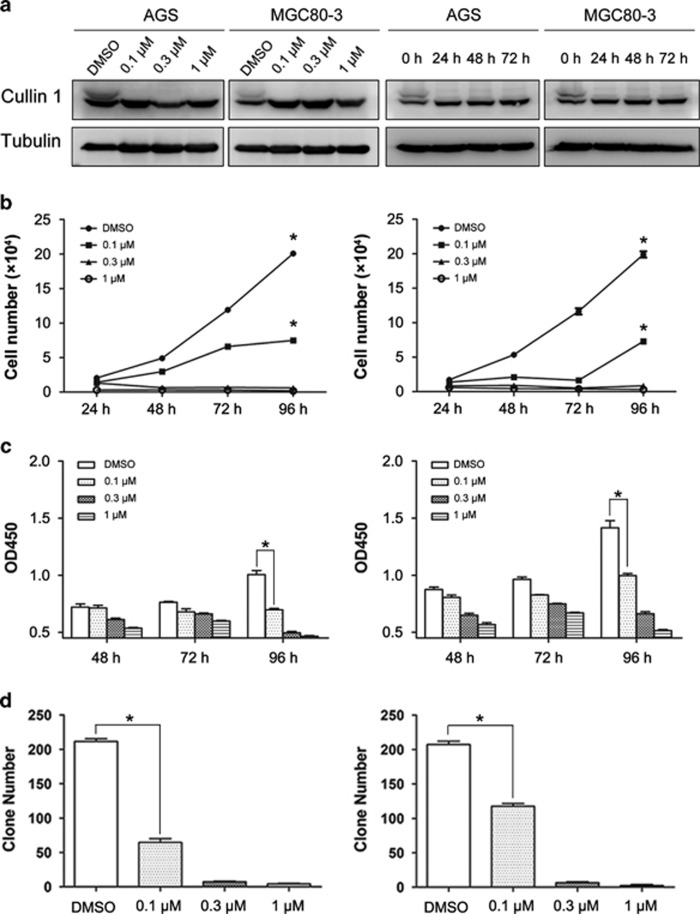
Growth inhibition of gastric cancer cells by MLN4924. (**a**) MLN4924 inhibited the neddylation of CUL1. AGS and MGC80-3 cells were treated under the indicated MLN4924 concentrations for 24–72 h, and then subjected to immunoblotting analysis of neddylation of CUL1. (**b**) MLN4924 suppressed the proliferation of gastric cancer cells. AGS and MGC80-3 cells were treated under the indicated MLN4924 concentrations for 24–96 h, then trypsinized and counted at each time point. (**c**) MLN4924 impaired the viability of gastric cancer cells. AGS and MGC80-3 cells were treated under the indicated MLN4924 concentrations and subjected to CCK8 colorimetric assay to assess their viability. (**d**) MLN4924 affected the colony-formation ability of gastric cancer cells. AGS and MGC80-3 cells were treated under the indicated MLN4924 concentrations for 9 days and then subjected to colony-formation assay. For panels **b** and **c**, *n*=3, and for panel **d**, *n*=4

MLN4924 is a neddylation inhibitor that was reported to be a potential therapeutic agent for several cancers, including leukemia and hepatocellular carcinoma.^27, 28^ However, its effect on gastric cancer cells is still unknown. Therefore, we determined the efficacy of MLN4924 in suppressing the growth of two gastric cancer cell lines (AGS and MGC80-3) by cell counting, CCK8 colorimetric assay and colony-formation assay. CUL1 neddylation was blocked by MLN4924 at 0.1 *μ*M, and this effect appeared only after 24h of treatment at 0.3 *μ*M (Figure 1a). The proliferation of AGS and MGC80-3 cells was significantly inhibited in a typical dose-dependent manner (Figures 1b–d, Supplementary Figure S1a). These results confirmed the impaired proliferation of gastric cancer cells by MLN4924.

### Cell cycle abnormalities were induced in gastric cancer cells by MLN4924

**Figure 2:**
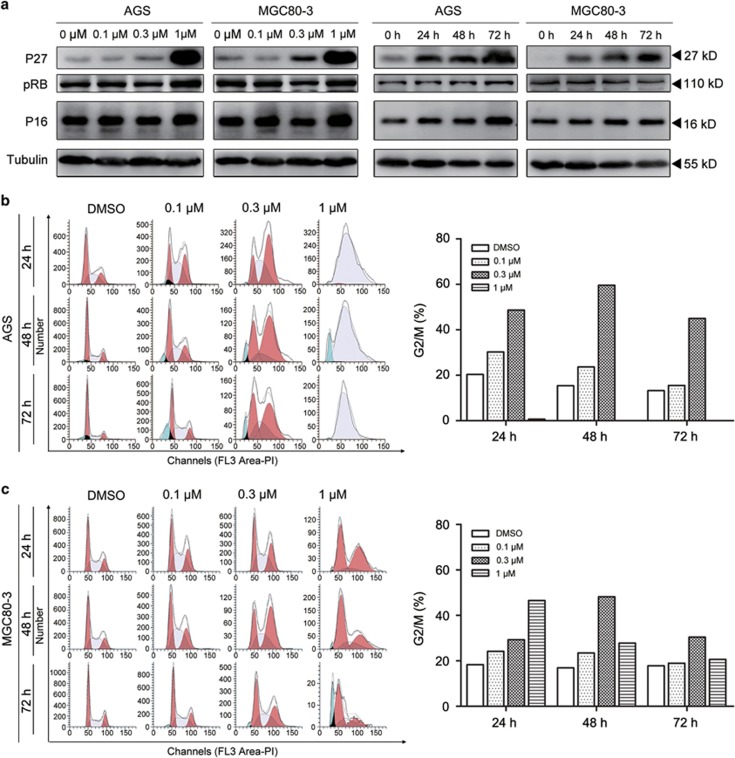
Cell cycle arrest of gastric cancer cells induced by MLN4924. (**a**) MLN4924 treatment led to the accumulation of senescence-associated proteins. AGS and MGC80-3 cells were cultured under the indicated MLN4924 concentration gradient for 72 h (left panel), or treated with 0.3 *μ*M MLN4924 and harvested at the indicated time point (right panel). The protein samples were subjected to immunoblotting analysis for the expression of P27, pRB, P16 and tubulin. (**b** and **c**) MLN4924 interrupted cell cycle progression at the G2/M phase. AGS and MGC80-3 cells treated with the indicated MLN4924 concentration, and subjected to propidium iodide staining and FCM to determine the cell cycle profile

It has been reported that MLN4924 stabilizes many SCF complex substrates, including cell cycle-related proteins. Consistent with previous results, we found that MLN4924 led to the accumulation of the SCF complex substrate P27. However, P16 from the INK4A family and its downstream effector RB were unaffected (Figure 2a). Accompanying the P27 upregulation, a large number of treated cells were observed to undergo a premature senescence compared with control groups (Supplementary Figure S1b). This observation suggested a critical role of P27 in MLN4924-induced growth inhibition.

Premature senescence is defined as a physiological mechanism that thwarts the proliferation of tumor cells by cell cycle arrest. Propidium iodide staining detected that the proportion of AGS cells arrested in G2/M phase increased as the concentration of MLN4924 was increased from 0.1 to 0.3 *μ*M, reaching the peak value at 48 h. However, cells treated with 1 *μ*M MLN4924 were restrained at S phase (Figure 2b). MGC80-3 cells shared a similar cell cycle alteration at 0.1 and 0.3 *μ*M MLN4924. However, when MGC80-3 cells were treated with 1 *μ*M, the proportion of G2/M-arrested cells reached the maximum more quickly at 24 h and then decreased over time, similar to the disappearance of the G2/M phase of AGS cells (Figure 2c).

P27 is well known for its functions in inhibiting CDK4/6-Cyclin D complexes and CDK2-Cyclin E/A complexes and blocking the cell cycle transition from the G1 to S phase. Therefore, although the treated cells were severely arrested in the G2/M phase instead of the G1 phase, we speculated that P27 promoted this cell cycle abnormality together with other cell cycle regulators.

### P27 silencing enhanced the suppression effects of MLN4924 on gastric cancer cells

**Figure 3:**
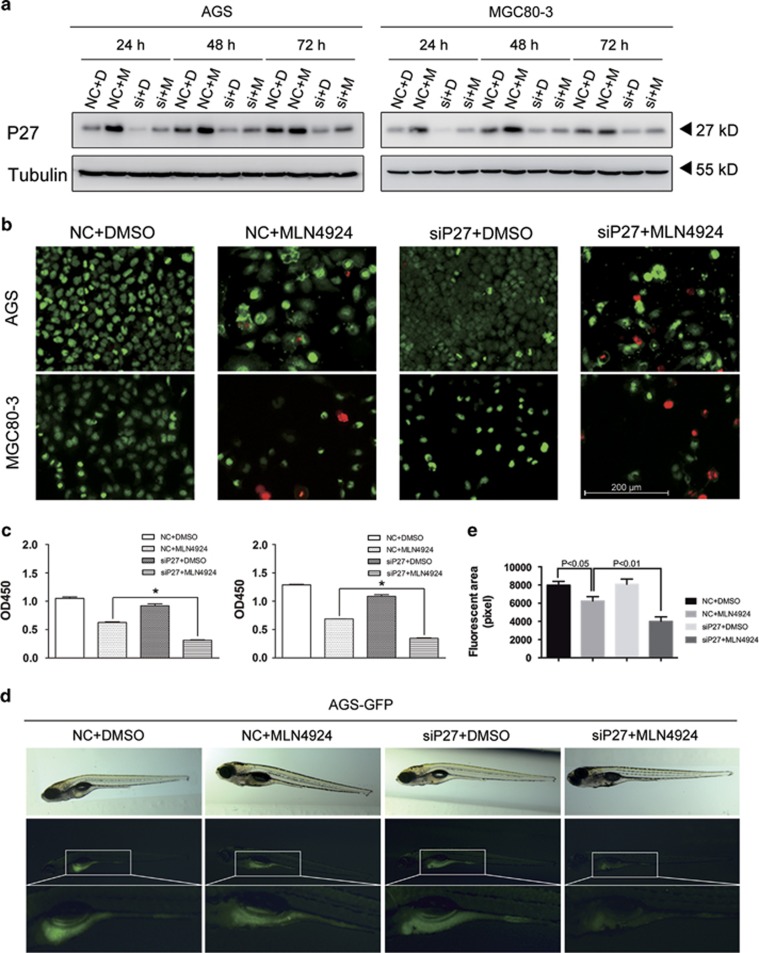
P27 silencing enhanced the cytotoxicity of MLN4924 in gastric cancer cells. (**a**) The designed siRNA downregulated P27 in gastric cancer cells. AGS and MGC80-3 cells were transfected with NC or siP27 for 72 h and then subjected to immunoblotting analysis for expression of P27 and tubulin. (**b** and **c**) SiP27 enhanced the cytotoxicity of MLN4924 in gastric cancer cells. AGS and MGC80-3 cells transfected with NC or siP27 were treated with DMSO or 0.3 *μ*M MLN4924. Cells were then subjected to stained by Live Dye (green) and propidium iodide (red) *in vivo* (bottom panel), or the CCK8 colorimetric assay (top panel). The dead cells (**b**) were indicated by red. (**d**) Smaller tumor size after combination treatment of the zebrafish xenograft models for AGS-GFP. AGS cells with GFP fluorescence transfected with NC or siP27 were transplanted into zebrafish embryos. Tumor sizes were indicated by the lateral fluorescent area. For panel **b**, *n*=4; for panel **d**, *n*=10. NC=Non-sense control siRNA; D=DMSO; M=MLN4924; si=siRNA for P27

To elucidate the functions of P27 in MLN4924-induced inhibition of proliferation, we knocked down P27 expression using two different siRNA in treated gastric cancer cells and effectively confined P27 accumulation (Figure 3a, Supplementary Figures S2a and S3a). Surprisingly, instead of obstructing the MLN4924-induced growth suppression, P27 silencing enhanced its cytotoxicity. As shown in the CCK8 assay, the cell viability of the combined treatment group was significantly lower than that of the group transfected with non-sense control sequence (NC) and treated by MLN4924 (Figure 3c,Supplementary Figure S3b). Similarly, in the colony-formation assay, the number of colonies in siP27-MLN4924 group was reduced by 17% compared with that of NC-MLN4924 group, although the number of colonies in the siP27-DMSO group was just slightly decreased (Supplementary Figure S2b). The Live-dead cell staining also suggested impaired cell viability in the siP27-MLN4924 group (Figure 3b, Supplementary Figure S2c). Besides, a similar phenomenon was recorded in HepG2 cells, a hepatocellular carcinoma cell line (Supplementary Figures S4a and b). These data demonstrated the significant enhancement of MLN4924 cytotoxicity after downregulation of P27 *in vitro*.

Furthermore, to assess the combined effects of MLN4924 treatment and P27 knockdown *in vivo*, we transplanted AGS-GFP (AGS cells expressing green fluorescent protein) which had been transfected with NC or siP27 into zebrafish embryos and incubated them with MLN4924.^29, 30^ This newly emerged zebrafish xenograft model provided a more direct way to observe the tumor growth, which enabled us to accurately determine the benefit of P27 knockdown after MLN4924 treatment.^31, 32, 33^ As shown in Figures 3d and e, the average tumor sizes of the four groups, indicated by the lateral fluorescent areas, had the same discrepancy between each other as we observed in *in vitro* studies.

### G2/M arrest and apoptosis induced by MLN4924 were augmented by siP27 in gastric cancer cells

**Figure 4:**
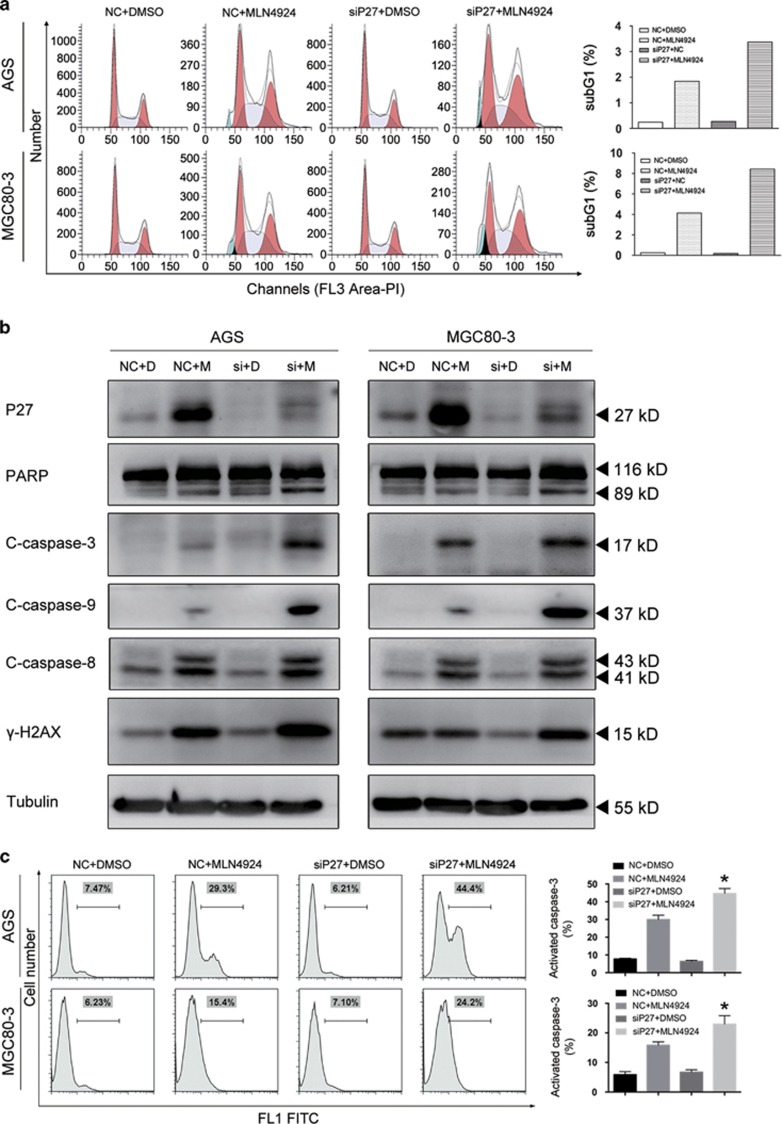
Intrinsic apoptotic signals induced by MLN4924 were strengthened by P27 silencing in gastric cancer cells. AGS and MGC80-3 cells transfected with NC or siP27 were treated with DMSO or 0.3 *μ*M MLN4924 for 72 h. (**a**) Sub-G1 accumulation following combination treatment. Cells were subjected to propidium iodide staining and FCM for analysis of cell cycle profile. (**b**) Expression of apoptotic proteins following combination treatment. Cells were subjected to immunoblotting analysis of the expression of the indicated proteins. (**c**) Enhanced caspase-3 cleavage by P27 silencing in MLN4924-treated cells. Cells were subjected to FITC-DEVD-FMK staining and FCM for cleaved caspase-3 quantification. For panel **c**, *n*=3. NC=Non-sense control siRNA; D=DMSO; M=MLN4924; si=siRNA for P27

Given the critical roles of P27 in the regulation of mitosis in human somatic cells, we initiated further cell cycle studies on the combined treatment groups. As shown in Figure 4a, G2/M arrest was strengthened in both AGS and MGC80-3 cells following simultaneous P27 silencing and MLN4924 treatment. This implied that P27 functioned as a protective factor against severe cell cycle abnormalities in these cells (Supplementary Figure S5). On the other hand, the sub-G1 proportions of the combined treatment group at 72 h rose to 1.8–2 times that of the NC-MLN4924 groups, suggesting that the increased cell death previously demonstrated was owing to enhanced apoptosis (Figure 4a, right panel). The activation of cleaved caspase-3 and PARP further confirmed this notion (Figure 4b). Flow cytometry (FCM) for cleaved caspase-3 showed a 9–14% augmentation of caspase-3 activation in the combined treatment group (Figure 4c). On the basis of these results, we proposed that P27 protects gastric cancer cells from the MLN4924-induced growth suppression, and that its functional deficiency would lead to the amplification of MLN4924 cytotoxicity.

In addition, while caspase-8 activation was only slightly enhanced in AGS cells, the cleavage of caspase-9 was increased in two combined treatment groups (Figure 4b,Supplementary Figure S3a). This discrepancy indicated that the intrinsic apoptotic pathway was the general mechanism for the enhanced apoptosis among different gastric cancer cell lines.^34^

### P27 silencing strengthened MLN4924-induced ROS overproduction

**Figure 5:**
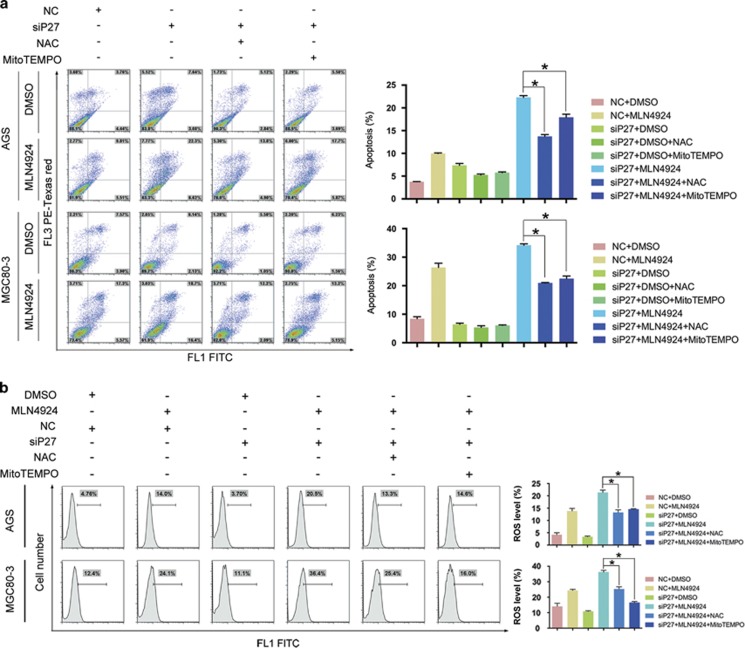
ROS overproduction following combination treatment resulted in augmented apoptosis. AGS and MGC80-3 cells transfected with NC or siP27 were treated under the indicated combination of MLN4924 and antioxidants. (**a**) The siP27-induced apoptosis augmentation in MLN4924-treated gastric cancer cells. The apoptosis was determined by Annexin V/propidium iodide double staining and FCM. (**b**) P27 silencing exacerbated ROS overproduction in MLN4924-treated cells. ROS levels were quantified by DCFH-DA staining and FCM. For panel **a** and **b**, *n*=3

Consistent with the intensive activation of caspase-3 and caspase-9 after the combination treatment, Annexin V/propidium iodide staining of AGS and MGC80-3 cells indicated an upregulated proportion of cells proceeding through apoptosis (Figure 5a, Supplementary Figures S6a and S7a). In previous works, MLN4924 has been shown to induce ROS overproduction in myeloid leukemia cells, which is tightly connected with the intrinsic apoptotic pathways.^26^ To determine whether ROS overproduction might be the central event for the augmented cytotoxicity caused by P27 silencing in the MLN4924 treatment, we used the fluorescent probe DCFH-DA to measure the ROS levels of the four groups at 24 h. ROS generation in groups treated with only MLN4924 was significantly higher than in the control groups, but it reached the highest level in the combined treatment groups (Figure 5b, Supplementary Figures S6b and S7b). As the antioxidant N-acetyl cysteine (NAC) ubiquitously suppressed ROS generation in the MLN4924-treated cells, MitoTEMPO (a specific scavenger of mitochondrial superoxide) exerted similar effects, suggesting mitochondria should be the major source of the accumulated ROS. In particular, ROS levels in the combined treatment groups were reduced to a comparable degree of those in the NC-MLN4924 groups. This reduction conferred an apparent resistance to the enhanced cytotoxicity in the gastric cancer cells and rescued them from excessive apoptosis (Figure 5a, Supplementary Figure S7a). The fact that NAC and MitoTEMPO shared similar protective effects toward the ROS-triggered apoptosis despite their different cellular compartment specificity emphasized the importance of mitochondria in enhanced apoptosis in the combined treatment groups.

### Enhanced exportation of apoptosis-related proteins from the mitochondria to the cytoplasm

Mitochondria are critical to basic cellular functions, and their dysregulation leads to disorders of respiration and energy supply which would guide cells to programmed death. Numerous substances inside the mitochondria need escape into the cytoplasm to trigger the downstream signals of apoptosis, such as caspase family activation and IAP family inhibition.^35, 36^

**Figure 6:**
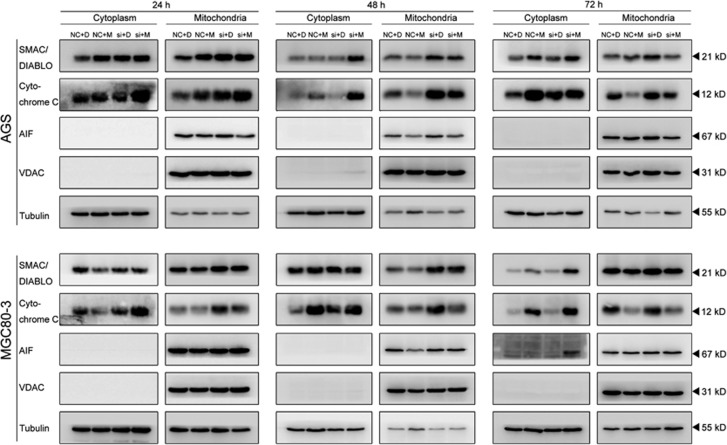
Translocation of apoptosis-related molecules from the mitochondrial intermembrane space to the cytoplasm in siP27-transfected gastric cancer cells. AGS and MGC80-3 cells transfected with NC or siP27 were treated with 0.3 *μ*M MLN4924 for 72 h, and subjected to subcellular fractionation and immunoblotting analysis for the subcellular localization of indicated proteins

We isolated mitochondria and cytoplasm from AGS and MGC80-3 cells to examine the export of three representative substances from mitochondria: apoptosis-inducing factor (AIF), IAP-binding mitochondrial protein (SMAC/DIABLO) and cytochrome c. As shown in Figure 6, at 24 h, AIF and SMAC/DIABLO did not redistribute between the two fractions in either cell line. However, cytochrome c in the combined treatment groups, the primary activator of caspase cascades, displayed a mild export into the cytoplasm. At 48 h in the AGS cells, higher levels of SMAC/Diablo and cytochrome c in this group were found in the cytoplasm. In contrast, only slight changes in cytochrome c were observed in the NC-MLN4924 group. In the MGC80-3 cells, only cytochrome c was exported into the cytoplasm after the MLN4924 treatment, indicating that apoptosis was initiated by P27 downregulation. In MGC80-3 cells, all three proteins including AIF had a significant escape from the intermembrane space of the mitochondrial membranes into the cytoplasm at 72 h. These results demonstrated that the synergistic effects of siP27 and MLN4924 were mediated by the typical intrinsic apoptotic pathway. The mitochondrion is the most pivotal compartment in this process, which connects the upstream signals from ROS overproduction with the following activation of executors for the apoptosis.

### P27 silencing deregulated mitochondrial membrane permeability by modulating the BCL-2 family

Under normal conditions, the permeability of the outer and inner membranes is critical for maintaining mitochondrial membrane potential (MMP). However, when cells undergo programmed cell death, the mitochondrial apoptosis-induced channels (MAC) are assembled and facilitate the export of cytochrome c, SMAC/DIABLO and AIF to activate their cytosolic effectors with the inevitable loss of MMP.^37, 38^

**Figure 7:**
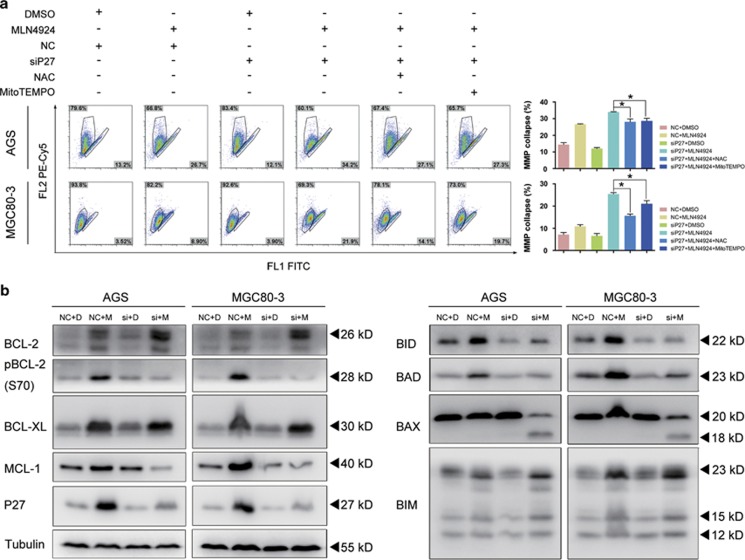
ROS triggered the collapse of MMP via siP27-induced profile alteration of BCL-2 family members in MLN4924-treated cells. (**a**) Deterioration of MMP collapse in the MLN4924-treated cells expressing low P27 was prevented by antioxidants. AGS and MGC80-3 cells transfected with NC or siP27 were treated under the combination of MLN4924 and antioxidants for 72 h, and subjected to JC-1 staining and FCM for the determination of MMP collapse. (**b**) P27 silencing led to alteration of BCL-2 family members expression in MLN4924-treated cells. AGS and MGC80-3 cells transfected with NC or siP27 were treated with 0.3 *μ*M MLN4924 for 72 h, and subjected to immunoblotting analysis for the detection of expression and status of indicated proteins. For panel **a**, *n*=3

In both gastric cancer cell lines treated with MLN4924, the proportions of cells undergoing the MMP collapse were significantly increased (Supplementary Figure S8). Meanwhile, the loss of MMP in the treated cells with lowered P27 expression was approximately 8–13% higher than that in the NC-MLN4924 groups (Figure 7a). Similarly, the Annexin V/propidium iodide staining showed that pre-incubation with NAC or MitoTEMPO was capable of rescuing cells from this augmentation (Figure 5a). This observation suggested that P27 silencing induced ROS overproduction and thus triggered MAC assembly and implied the robust ROS production might either recruit the key components of MAC from the pro-apoptotic BCL-2 family or repress their regulators from the anti-apoptotic BCL-2 family.

Western blotting demonstrated that several members from the pro-apoptotic BCL-2 family were upregulated in the MLN4924-treated groups, including BIM, BID and BAD (Figure 7b). Moreover, the expression of BCL-2, BCL-XL and MCL-1, which belong to the anti-apoptotic BCL-2 family were strongly increased in the combined treatment groups. The phosphorylation of BCL-2 was also significantly augmented. This posttranscriptional modification has been reported to promote cellular survival under ROS overproduction crisis. In contrast, expression of MCL-1 in the combined treatment groups remarkably decreased while BCL-XL was unaffected compared with the NC-MLN4924 groups. As for BCL-2, paradoxical changes of its protein level and phosphorylation state were observed in both cell lines. While its protein level was significantly increased, the phosphorylation of BCL-2, which is closely connected with P27 overexpression and ROS suppression, was severely reduced to the basal level.^39^ Correspondingly, in the pro-apoptotic family, the activation of BIM was further enhanced, even though the protein levels of BID and BAD were reduced. Most importantly, silencing P27 during MLN4924 treatment induced an unexpected cleavage of BAX into p18-BAX. This transformation of BAX has been reported to enable itself to avoid the BCL-2 inhibitory functions and directly construct MAC.^40^

Therefore, we concluded that P27 silencing in the MLN4924-treated cells activated the assembly of MAC components via ROS overproduction and further suppressed the major inhibitors by interfering with their protein levels, blocking their phosphorylation or directly sequestering them, thus strengthening the apoptotic signals in the gastric cancer cells.

## Discussion

As a critical regulator of cell cycle progression, P27 has been considered to be a tumor-suppressor gene in the conventional view. It is degraded by the SCF-complex-mediated ubiquitin-proteasome system. However, the functions of P27 as an important ubiquitin E3 ligase substrate remain unclear. In this study, we provided new insights into the functions of p27, which suggested that p27 inhibition could be considered as a potential treatment for cancer, particularly in combination with MLN4924.

When cells are preserved in the quiescent state, P27 is upregulated to block uncontrolled cell cycle progression. By induced fit, P27 can bind with the three different CDK-Cyclin complexes, CDK4/6-CyclinD, CDK2-CyclinE/A and CDK1-CyclinB, and impede their kinase activities. Thereby, P27 suppresses the major facilitators of the G1/S transition and prevents the cell cycle from entering subsequent phases. In our study, the discrepancy between cell cycle arrest at different concentration of MLN4924 suggests that P27 coordinated with other regulators to determine cell cycle progression. When P27 expression was silenced first, the proportion of G2/M-arrested cells, which should have reached the peak value with 0.3 *μ*M MLN4924 treatment, strikingly increased without additional drugs. This finding is in accordance with previous studies about the central position of P27 in the regulation of the G1 phase^11^ and implies that it has protective potential for gastric cancer cells. Intriguingly, we also established the improved efficacy of MLN4924 by P27 knockdown in *in vivo* studies based on the zebrafish xenograft model,^29, 33^ and found this sensitization by P27 silencing might be applied in other cancers as well (Supplementary Figure S4). These discoveries provided the proof-of-concept evidences for the application of P27 inhibition in cancer treatment, especially with MLN4924.

As an important initiator for MAC component activation,^41^ ROS overproduction has been detected previously in MLN4924-treated leukemia cells in others' studies.^28^ In this work, after silencing P27, the MLN4924-treated cells underwent increased ROS generation together with an augmented MMP collapse and apoptosis. The NAC's antagonism to this cytotoxicity sensitization explicitly verified ROS as the initiator in P27-related apoptotic signal pathways. A similar effect of MitoTEMPO, the mitochondrion-specific ROS scavenger, indicated that the mitochondria were the major source of the excessive ROS production in the MLN4924-treated cells with lower P27. In addition, the simultaneous translocation of cytochrome c, SMAC/DIABLO and AIF in the combined treatment groups (Figure 6) also emphasized the critical role of mitochondria for cytotoxicity sensitization of MLN4924 and indicated that the assembly of MAC might be the pivotal process that connected P27 with the downstream signals for apoptosis.

Conforming to previous reports that robust ROS production activates the mitochondrial outer membrane pore assembly, the protein pattern shift of BCL-2 family members was observed after P27 knockdown in treated gastric cancer cells. In MLN4924-treated cells, the anti-apoptotic family members were significantly upregulated, while BAK and BAX, the major components of MAC, remained unchanged. In contrast, after P27 knockdown, the MCL-1, BCL-2 phosphorylation, BAX cleavage and BIM in the treated cells behaved synergic to augment apoptosis (Figure 7b). BCL-2 phosphorylation has been shown to upregulate P27 and resist intracellular ROS-induced damages in the previous study. However, our results suggested that P27 was also able to induce the BCL-2 phosphorylation for apoptotic resistance, which therefore revealed a mutual activation between P27 and BCL-2 phosphorylation for cell survival. As for BAX cleavage, it was mediated by the calcium-dependent protease calpain and resulted in the formation of p18-BAX, which was not subjected to inhibition by BCL-2.^40, 42, 43^ As calpain is the key executor of the non-ubiquitin degradation of P27 in the cytoplasm,^44^ P27 that accumulated after MLN4924 treatment might protect cells from the uncontrolled p18-BAX by saturating the enzymatic activity of calpains.

In previous studies, the sub-cellular translocation of P27 with different phosphorylation status was regarded as the decisive point for the susceptible dual roles of P27 in tumorigenesis. The threonine-187-phosphorylated (T187-phosphorylated) P27 is the candidate substrate for SCF^SKP2^-mediated proteasome degradation in the nucleus, while the phosphorylation at threonine-157, threonine-198 and serine-10 targets P27 into the cytoplasm and impairs the function of its nuclear localization sequence,^45, 46, 47^ providing the basis for its cytoplasmic degradation by the Kip1 ubiquitination-promoting complex (KPC) and calpain. In theory, SCF^SKP2^ inhibition by MLN4924 should lead to the accumulation of T187-phosphorylated P27, which is restricted in the nucleus. This deduction contradicted the apoptotic signals that emerged in our study that required the direct interaction of P27 in the cytoplasm, such as the ROS overproduction and BAX cleavage. Because the two types of phosphorylation mainly decide the sub-cellular localization of P27 and are inconvertible to each other, we assumed that there must be a recycling mechanism that relieves T187 phosphorylation of P27 and prepares it for cytoplasm-targeted phosphorylation by other kinases, such as AKT and MEK. Further analysis, as well as more specific interfering methods for the different phosphorylation events, are necessary to test this assumption.

Even though several studies have reported a susceptible oncogenic character of P27 in tumors treated by preussin or proteasome inhibitors, they have not clarified the underlying mechanism.^48, 49, 50^ Our study for the first time revealed P27 functioned as a protective protein for tumor cells by suppressing ROS generation, modulating the profile of BCL-2 family members and maintaining the normal mitochondrial status in gastric cancer cells treated with MLN4924. In addition, the remarkable sensitization of MLN4924 by P27 silencing revealed in our study indicates that there is a potential of combining MLN4924 with P27 inhibitors to improve its therapeutic efficacy.

## Materials and Methods

### Cell culture and reagents

The human gastric cancer cell lines AGS and MGC80-3 were obtained from the Type Culture Collection of the Chinese Academy of Sciences (Shanghai, China). These cells were cultured in Dulbecco's modified Eagle's medium (Gibco, Life Technologies, Carlsbad, CA, USA) containing 10% fetal bovine serum and 1% penicillin-streptomycin at 37 °C with 5% CO_2_. MLN4924 was purchased from Millennium (Cambridge, MA, USA). For *in vitro* studies, MLN4924 was stored at a concentration of 10 *μ*M at −20 °C. NAC and MitoTEMPO were purchased from Sigma (St. Louis, MO, USA).

### Immunoblotting

Cell lysates were extracted with cell lysis buffer (Beyotime Biotechnology, Nantong, China), and the protein concentration in the lysates was quantified using a Micro BCA Protein Assay Kit (Sangon Biotech, Shanghai, China). A total of 20–50 *μ*g of each cell lysate sample was loaded for immunoblotting and detected by antibodies that recognize Tubulin, VDAC, AIF, SMAC/DIABLO, cytochrome c (Epitomics, Hangzhou, China), CUL1 (Santa Cruz Biotechnology, Santa Cruz, CA, USA), CDKN1B/P27, CDKN2A/P16, PARP, γH2AX, caspase3, caspase8, caspase9, BCL-2, pBCL-2 (S70), BCL-XL, MCL-1, BID, BAD, BAX, and BIM (Cell Signaling Inc., Danvers, MA, USA).

### RNA interference

siRNA oligonucleotides were synthesized by GenePharma (Shanghai, China) and were transfected into gastric cancer cells by Lipofectamine 2000 (Invitrogen, Carlsbad, CA, USA). Briefly, siRNA and Lipofectamine 2000 were incubated separately with Opti-MEM (Invitrogen, Carlsbad, CA, USA) for 5 min and then mixed together for 20 min at room temperature. The mixture was then applied to the cells plated in 60-mm dishes (final concentration of siRNA is 50 nM). The sequence of the siRNA for CDKN1B/P27 (siP27) was 5′-GCAACCGACGAUUCUUCUATT-3′. The scrambled siRNA control (siControl) sequence was 5′-TTCTCCGAACGTGTCACGTTT-3′.

### Propidium iodide staining and FACS analysis

For cell cycle distribution and apoptosis analysis, cells were harvested and fixed in 70% ethanol at −20 °C overnight. They were then incubated with the staining buffer containing 36 *μ*g/ml propidium iodide (Sigma) and 400 *μ*g/ml RNase (Roche, Mannhein, Germany) at 37 °C for 20 min and analyzed by FCM (CyAnTM ADP, Beckman Coulter, Brea, CA, USA). Data were analyzed with ModFit LT software (Verity Software House, Topsham, ME, USA).

### SA-β-galactosidase staining

Intact cells or those transfected with siP27 for 24 h were split and seeded into 12-well plates at 10 000 cells per well and then evaluated by SA-β-gal staining as described previously.^51^

### Cell proliferation and colony-formation assay

Cells were either seeded into 24-well plates at 6000 cells per well in triplicate for cell counting at indicated time points, or they were seeded into 96-well plates at 1000 cells per well in quadruplicate for the CCK8 colorimetric assay (Dojindo, Kumamoto, Japan) according to the manufacturer's specifications. For the colony-formation assay, the split cells were seeded into 6-well plates (500 cells per well) and cultured for 10 days. The colonies on the plates were fixed with 4% paraformaldehyde and then stained with crystal violet.

### Viability staining

The P27-silenced cells were seeded into 24-well plates at 10 000 cells per well and incubated with DMSO or 0.3 *μ*Mol/l MLN4924 for 72 h. They were then washed with PBS and incubated with 500 *μ*l staining buffer containing 0.5 *μ*l Live Dye (Enzo Life Sciences, Farmingdale, NY, USA) and 0.5 *μ*l propidium iodide (2.5 mg/ml, Enzo Life Sciences) for 20 min in a 37°C incubator with 5% CO_2_. The cells were photographed under a fluorescence microscope (Leica, Wetzlar, Germany).

### Establishment of AGS-GFP stable cell lines

AGS cells expressing GFP were established as described. Briefly, cells were seeded in 100-mm dishes and transfected with the lentivirus containing GFP DNA. Cells with GFP fluorescence were selected by MoFlo XDP Cell Sorter (Beckman Coulter) and cultured in complete cell culture medium containing puromycin (Beyotime Biotechnology) at 0.5 *μ*g/ml.

### Zebrafish xenograft model

The fertilized wild-type AB zebrafish eggs were collected and incubated at 29°C in E3 embryo medium for 48 h. At 48 h.p.f. (hour post fertilization), the chorion were removed by forceps. The embryos were then treated with 0.02 mg/ml tricaine (Sigma) before receiving microinjection of cancer cells with GFP. One hundred and fifty cells transfected with NC or siP27 were transplanted into the space between the periderm and the yolk syncytial layer. Thirty injected embryos were incubated with 1 *μ*M MLN4924 diluted in E3 embryo medium for 96 h. Ten zebrafishes were randomly selected and photographed under a fluorescence microscope (Leica). The lateral fluorescent area indicating the size of the tumor mass was calculated and analyzed by ImageJ software.

### Quantification of cleaved caspase-3

To measure caspase-3 cleavage, cells were harvested and incubated with the FITC-DEVD-FMK (BioVision, Milpitas, CA, USA) in PBS in a 37°C incubator with 5% CO_2_ for 30 min. Samples were centrifuged at 3000 r.p.m., and the supernatant was removed. The cells were resuspended in wash buffer and centrifuged twice. Cells were re-suspended in PBS, and caspase-3 cleavage was analyzed by FCM.

### Detection of ROS and measurement of MMP

For the ROS detection, cells were harvested and mixed with 10 *μ*M DCFH-DA (Beyotime Biotechnology) diluted in PBS at 37°C for 20 min. For the positive control groups, cells were incubated with 0.05 mg/ml Rosup for 20 min first and then with DCFH-DA. Cells were washed twice, followed by analysis by FCM. The same method was also applied to mitochondria potential monitoring with the probe JC-1 (Beyotime Biotechnology) and the positive control groups were incubated with 10 *μ*M CCCP instead. Green (510–530 nm) and Red (650 nm) fluorescence emission from cells was measured following excitation at 488 nm.

### Mitochondria isolation

Gastric cancer cells were trypsinized, and washed by PBS. The cell suspension was centrifuged at 600 × *g* at 4°C for 5 min and the supernatant was discarded. The mitochondria isolation medium (Beyotime Biotechnology) containing 1 *μ*M PMSF was then added to re-suspend the cell pellets in proportions of 1 *μ*l per 10^4^ cells. The cells were homogenized, and the supernatant was retained following a 10 min 600 × *g* centrifugation at 4°C. The precipitate of mitochondria was isolated after 11 000 × *g* centrifugation for 10 min. The supernatant were again centrifuged at 12 000 × *g* for 10 min. After discarding the precipitate, the leftover was preserved as the cytoplasm protein sample.

### Statistical analysis

The statistical significance of differences between groups was assessed using GraphPad Prism5 software. The unpaired two-tailed *T* test was used for the comparison of parameters between two groups. For all the tests, three levels of significance (*P*<0.05, *P*<0.01 and *P*<0.001) were used. *P<0.01.

## Abbreviations

CDKN1B: Cyclin-dependent kinase inhibitor 1B
SCF complex: SKP2-Cullin1-F-Box Complex
GFP: green fluorescent protein
ROS: reactive oxygen species
AIF: apoptosis-inducing factor
KPC: Kip1 ubiquitination-promoting complex
MMP: mitochondrial membrane potential
MAC: mitochondrial apoptosis-induced channel

## Notes

The authors declare no conflict interest.
