# Supplementary material for: The novel protective role of P27 in MLN4924-treated gastric cancer cells
Source: Cell Death Dis. 2015 Aug 27;6(8):e1867–. doi: 10.1038/cddis.2015.215 (PMC4558507; doi:10.1038/cddis.2015.215)
Supplement: Supplementary Figure Legends [file cddis2015215x1.pdf]

Supplementary Figure Legends

Supplementary Figure 1. MLN4924 affected the colony formation ability of gastric cancer cells and induced premature senescence. (a and b) AGS and MGC80-3 cells were treated under the indicated MLN4924 concentration gradient ,then subjected to colony formation assay **(a)** or the senescence associated β-gal staining **(b)**.

Supplementary Figure 2. P27 silencing reinforced MLN4924-induced impairment of the viability of gastric cancer cells. (a) AGS and MGC80-3 cells were transfected with NC or siP27 for 72 h and then subjected to IB analysis for expression of P27 and Tubulin. **(b and c)** AGS and MGC80-3 cells transfected with NC or siP27 were treated with DMSO or 0.3 μM MLN4924, and subjected to colony formation assay or stained by Live Dye (green) and PI (red) *in vitro*. The dead cells were indicated by the red color. For panel b, n=3.

Supplementary Figure 3. P27 silencing by another siRNA augmented the cytotoxicity of MLN4924 on gastric cancer cells. (a) AGS and MGC80-3 cells transfected with NC or siP27-2 were treated with DMSO or 0.3 μM MLN4924 for 72 h and then subjected to IB analysis of the expression of P27 and Tubulin. **(b)** AGS and MGC80-3 cells transfected with NC or siP27-2 were treated with DMSO or 0.3 μM MLN4924 from 24 h to 72 h. Their proliferation curves were plotted by the CCK8 assay. For panel b, n=4. NC = Non-sense control siRNA; D = DMSO; M = MLN4924; si-2 = siP27-2.

Supplementary Figure 4. P27 silencing enhanced the MLN4924-induced PARP cleavage and growth inhibition in HepG2. (a and b) HepG2 cells transfected with NC or siP27 were treated with DMSO or 0.3 μM MLN4924 for 72 h, and subjected to IB analysis of PARP cleavage, P27 and Tubulin **(a)**, or to CCK8

assay **(b)**. For panel b, n=4. NC = Non-sense control siRNA; D = DMSO; M = MLN4924; si = siRNA for P27.

Supplementary Figure 5. Increased G2/M arrest in cells treated with siP27 and MLN4924 together. AGS and MGC80-3 cells transfected with NC or siP27 were treated with DMSO or 0.3 μM MLN4924 for 72 h, and subjected to PI staining and FCM for analysis of cell cycle profile.

Supplementary Figure 6. The effects of NAC and MitoTEMPO on the basal level of apoptosis and ROS in gastric cancer cells. AGS and MGC80-3 cells which were transfected with NC or siP27 were treated under the indicated conditions. **(a)** The apoptosis was determined by Annexin V/PI double staining and FCM. **(b)** The ROS level was quantified by DCFH-DA staining and FCM. For panel a and panel b, n=3.

Supplementary Figure 7. P27 knockdown by siP27-2 enhanced the apoptosis induced by MLN4924 by triggering ROS overproduction. AGS and MGC80-3 cells which were transfected with NC or siP27-2 were treated under the indicated combination of MLN4924 and antioxidants. **(a)** The apoptosis was determined by Annexin V/PI double staining and FCM. **(b)** The ROS level was quantified by DCFH-DA staining and FCM. For panel a and panel b, n=3.

Supplementary Figure 8. Deterioration of MMP collapse in MLN4924-treated cells with low P27. AGS and MGC80-3 cells which were transfected with NC or siP27 were treated with DMSO or 0.3 μM MLN4924 for 72 h, and subjected to JC-1 staining and FCM to determine MMP collapse.
