# Supplementary figures and images for: The novel protective role of P27 in MLN4924-treated gastric cancer cells
Source: Cell Death Dis. 2015 Aug 27;6(8):e1867–. doi: 10.1038/cddis.2015.215 (PMC4558507; doi:10.1038/cddis.2015.215)

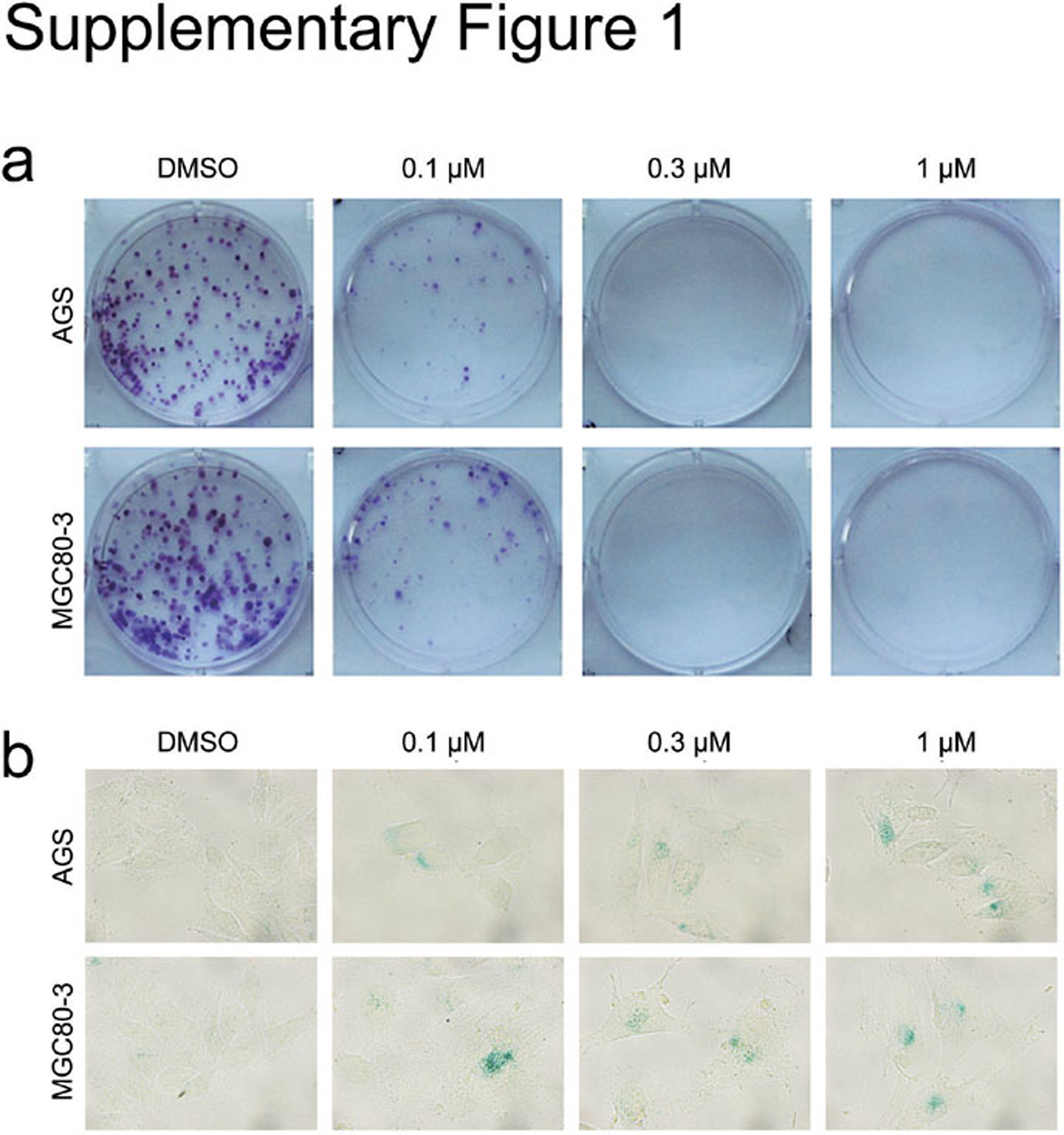

Supplement: Supplementary Figure 1 [file cddis2015215x2.tif]

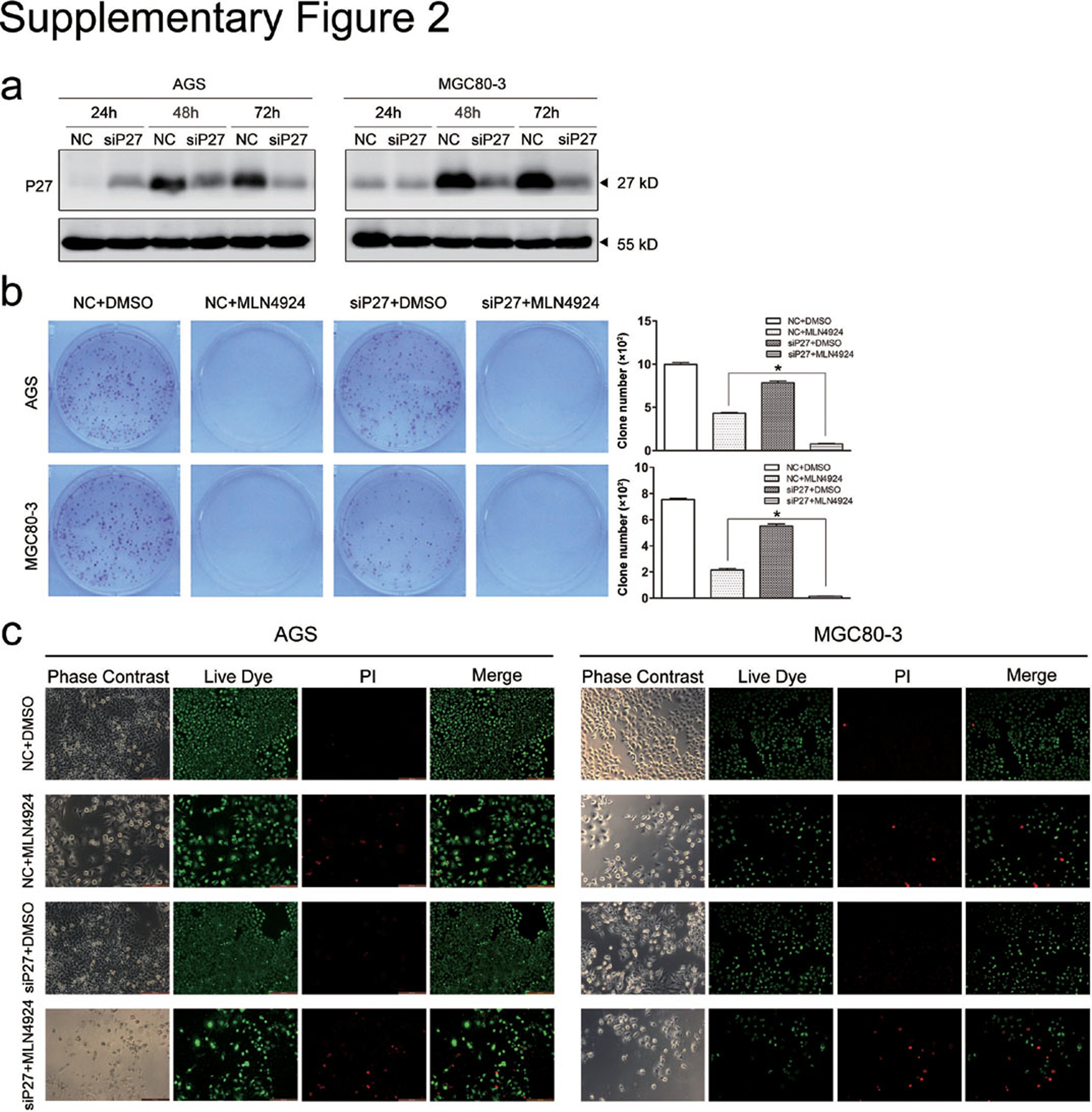

Supplement: Supplementary Figure 2 [file cddis2015215x3.tif]

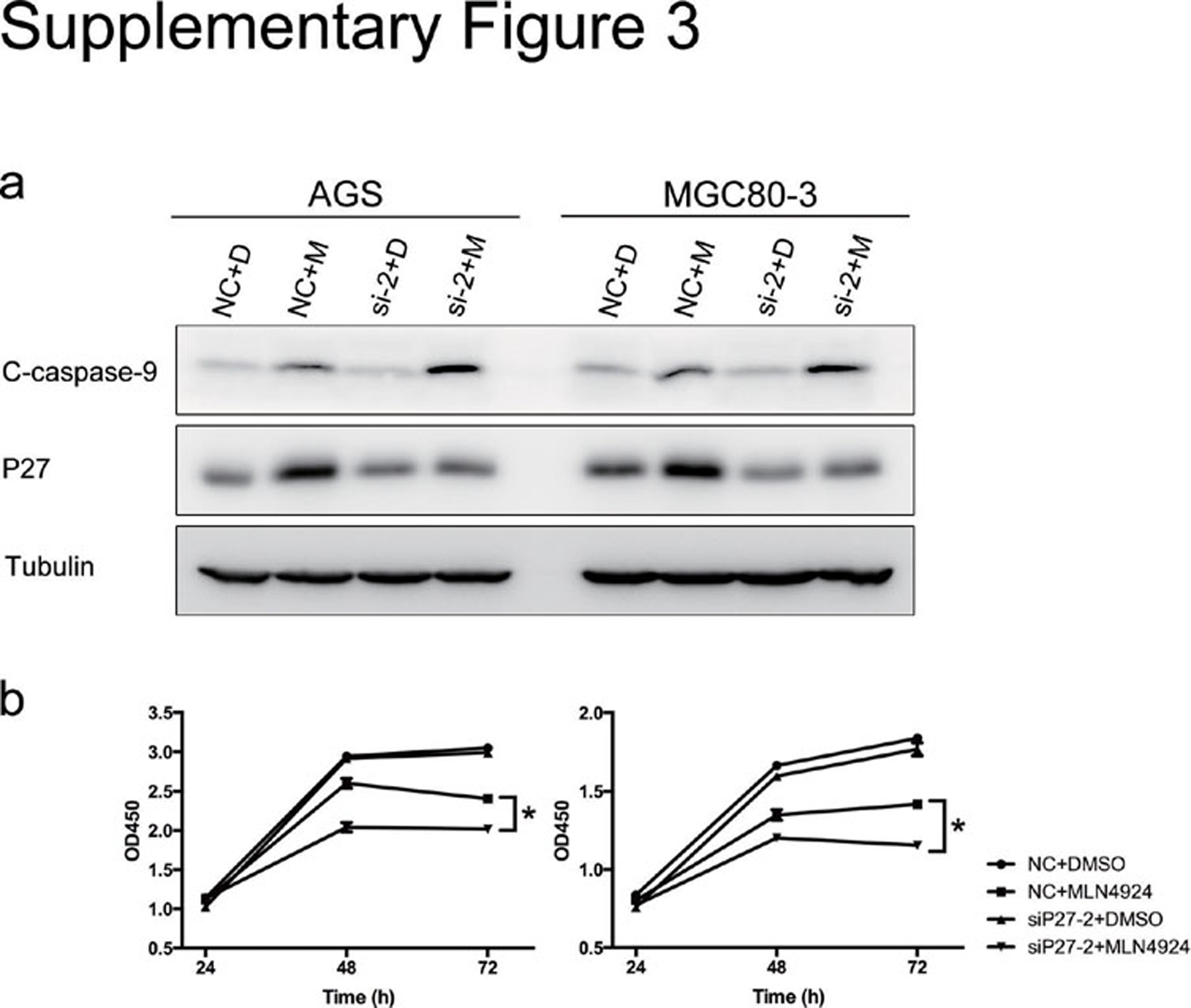

Supplement: Supplementary Figure 3 [file cddis2015215x4.tif]

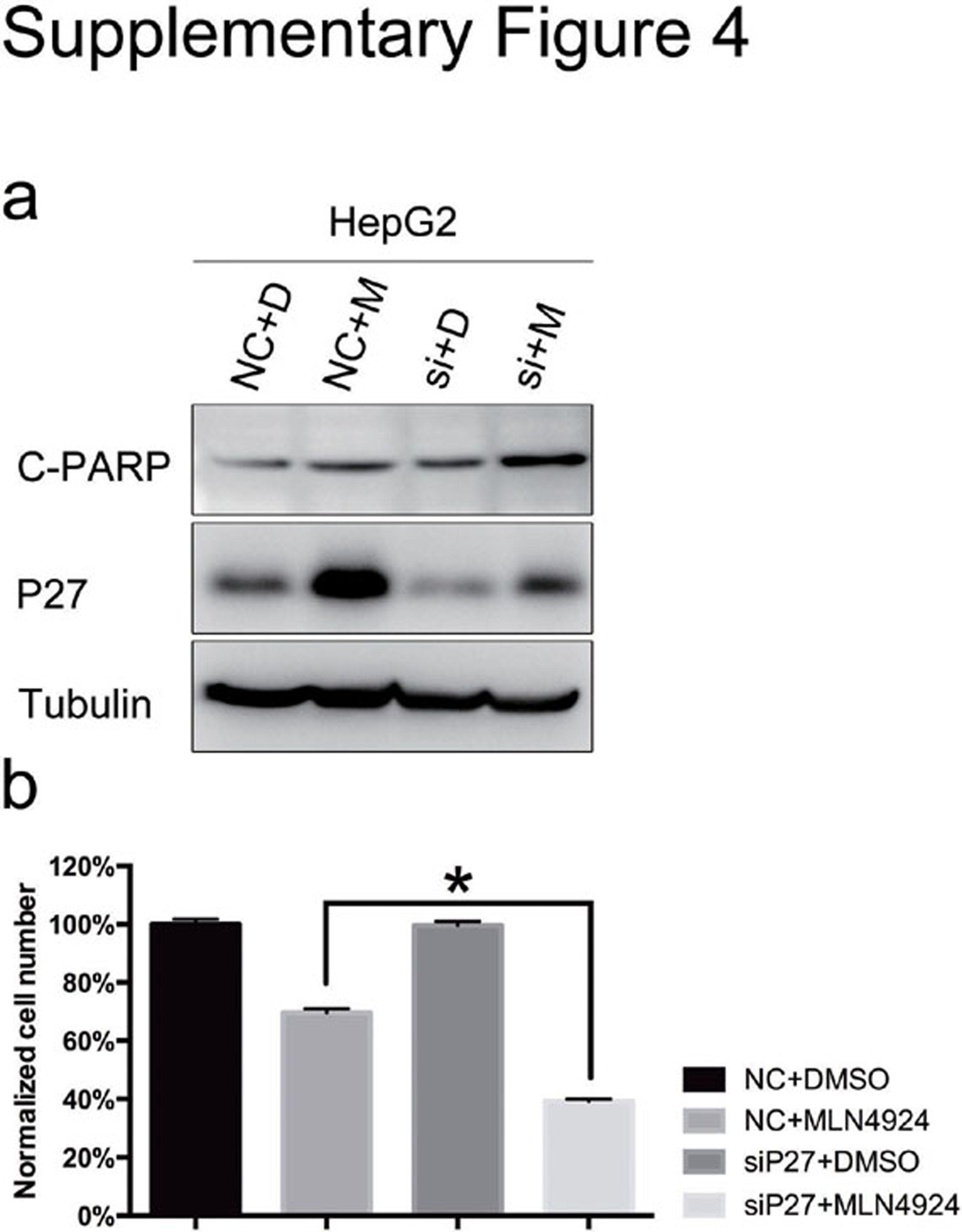

Supplement: Supplementary Figure 4 [file cddis2015215x5.tif]

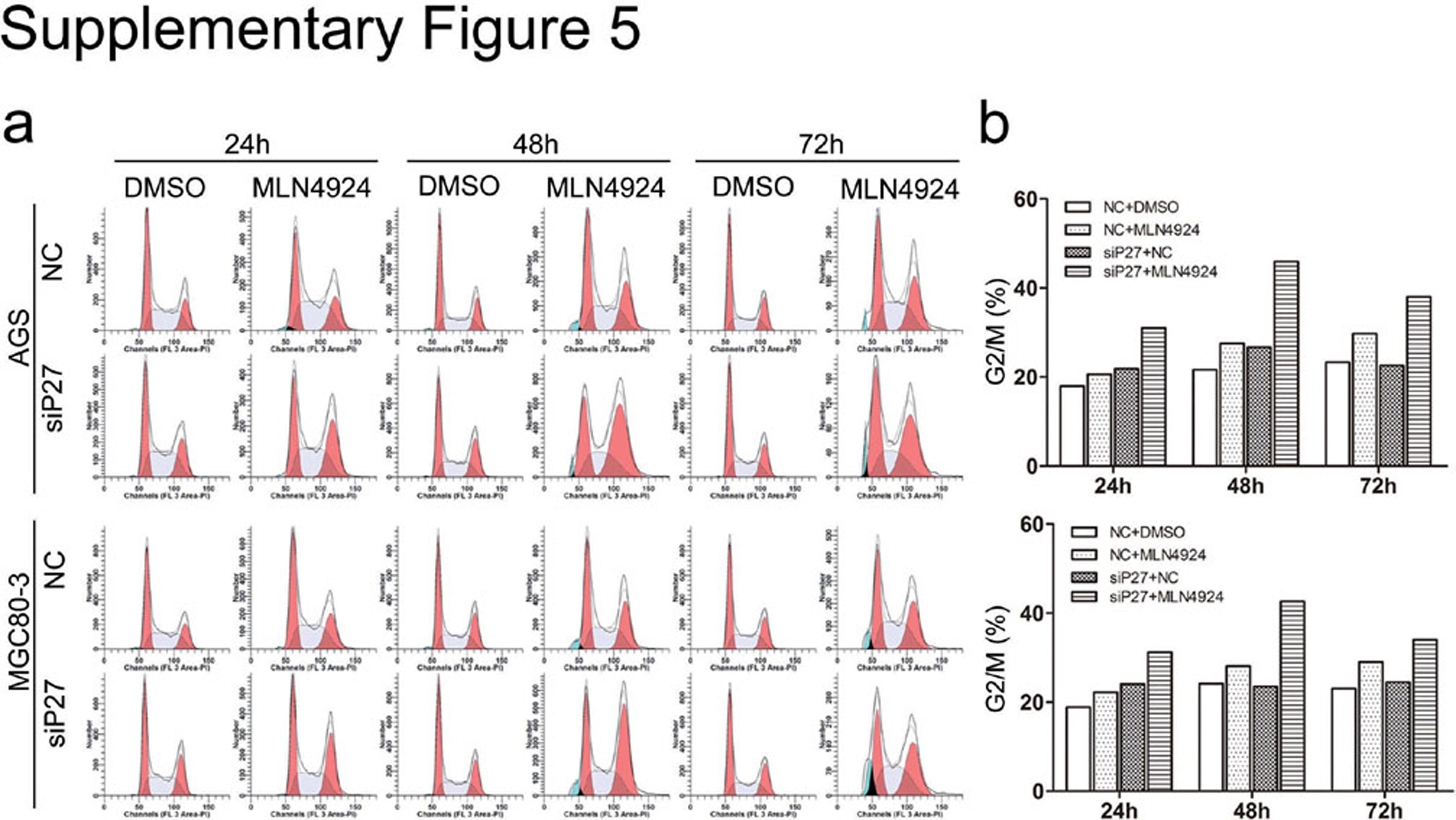

Supplement: Supplementary Figure 5 [file cddis2015215x6.tif]

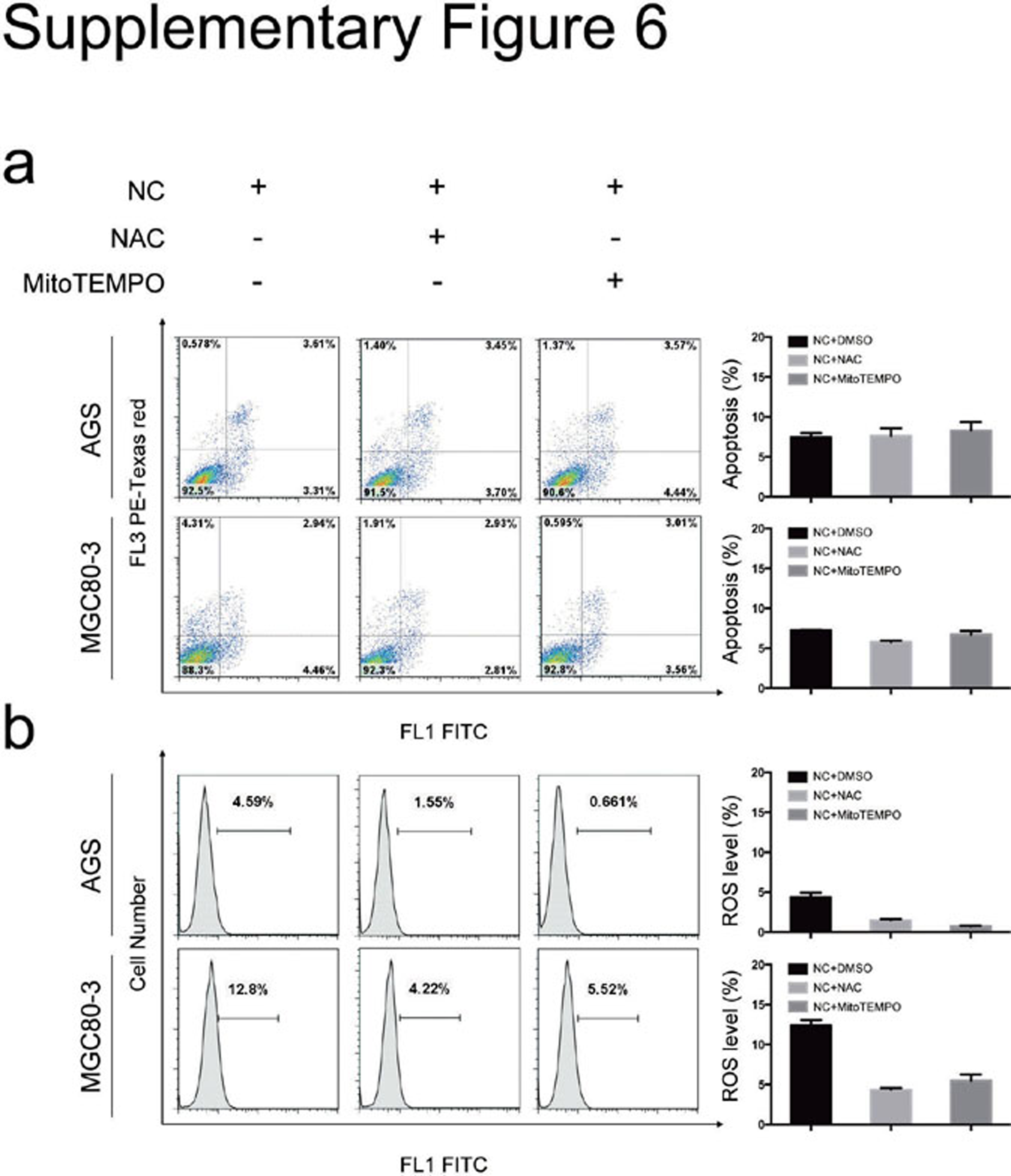

Supplement: Supplementary Figure 6 [file cddis2015215x7.tif]

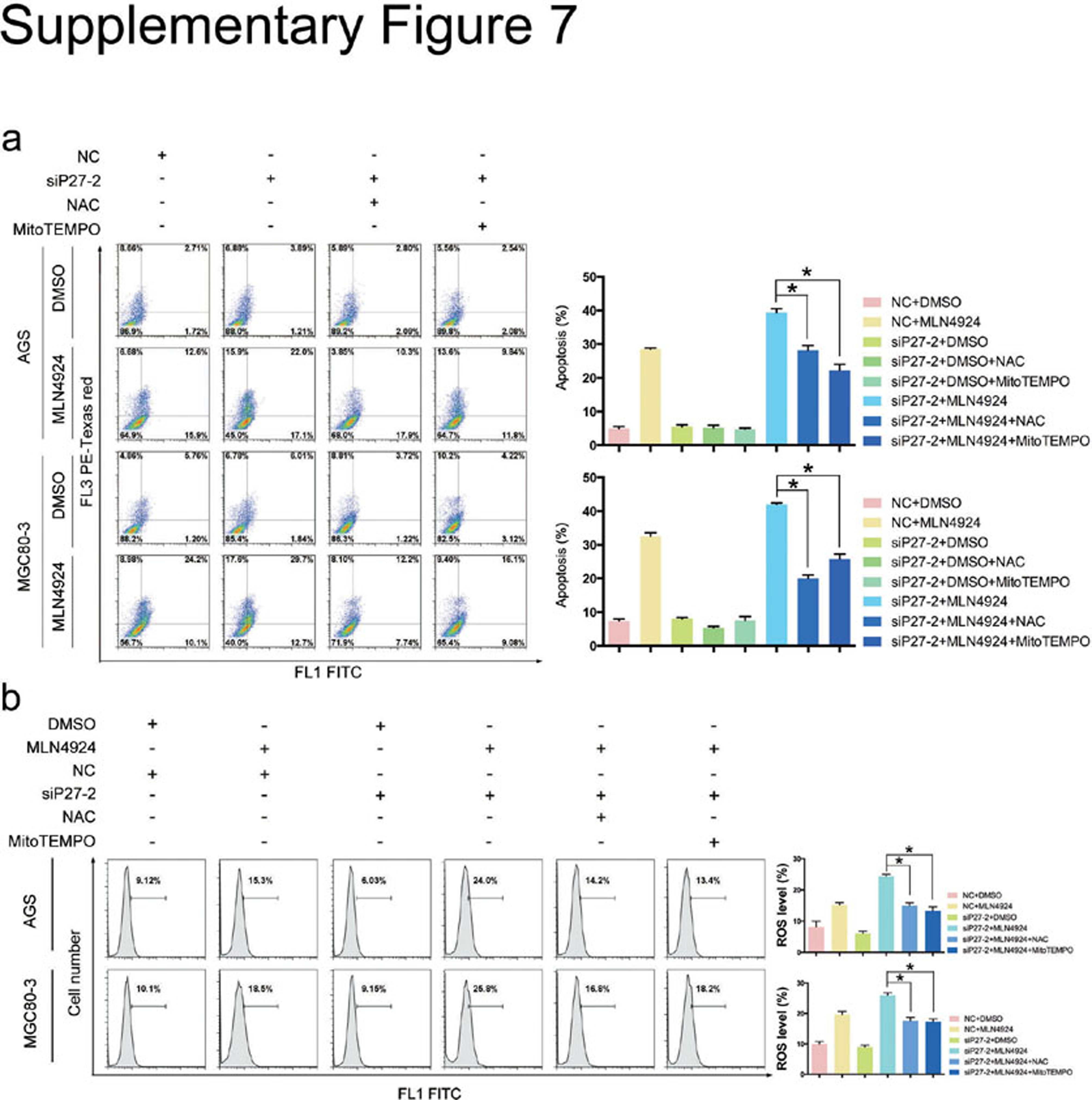

Supplement: Supplementary Figure 7 [file cddis2015215x8.tif]

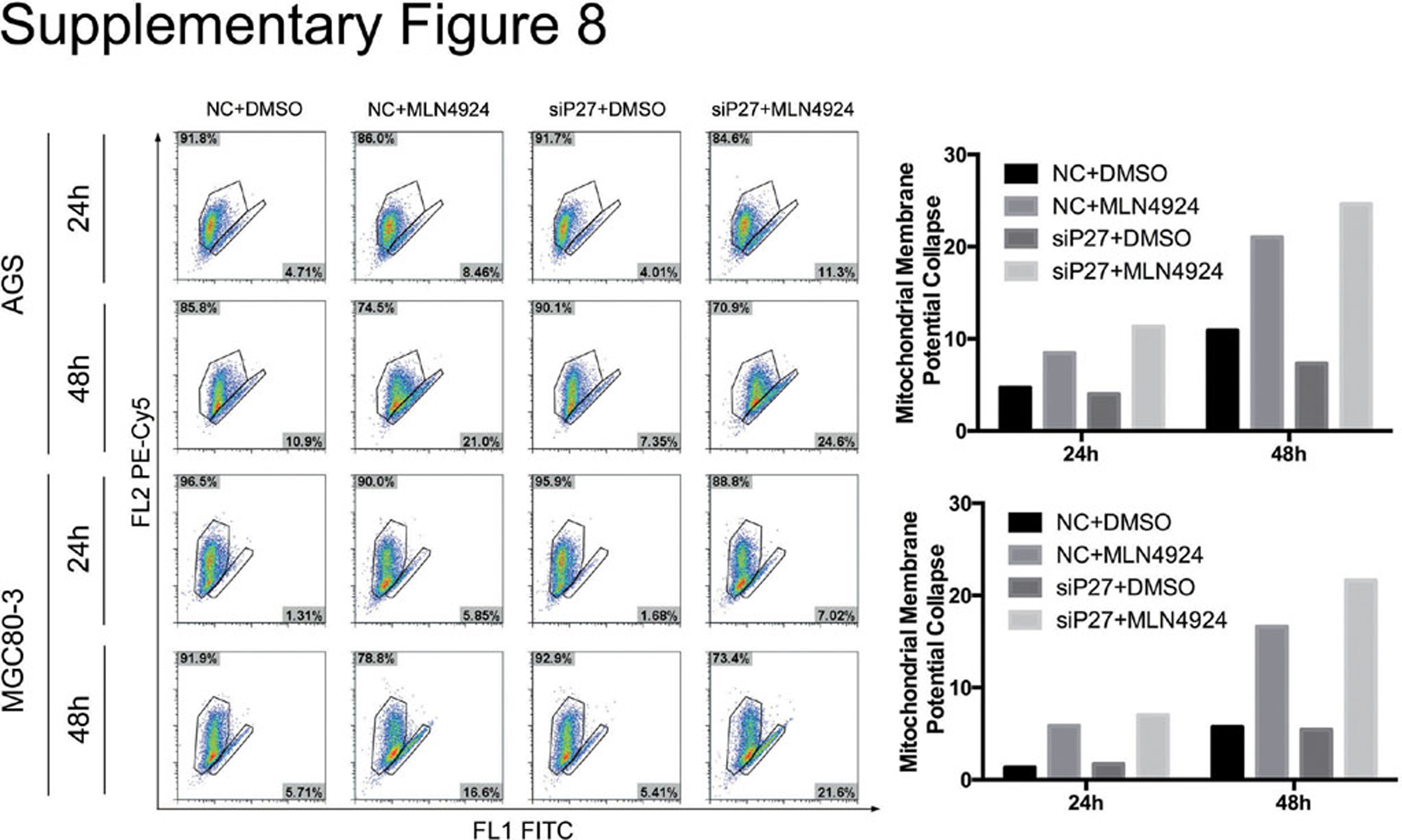

Supplement: Supplementary Figure 8 [file cddis2015215x9.tif]
